# Calcium signals can freely cross the nuclear envelope in hippocampal neurons: somatic calcium increases generate nuclear calcium transients

**DOI:** 10.1186/1471-2202-8-57

**Published:** 2007-07-30

**Authors:** Anja Eder, Hilmar Bading

**Affiliations:** 1 Department of Neurobiology, Interdisciplinary Centre for Neurosciences, Im Neuenheimer Feld 364, 69120 Heidelberg, Germany

## Abstract

**Background:**

In hippocampal neurons, nuclear calcium signaling is important for learning- and neuronal survival-associated gene expression. However, it is unknown whether calcium signals generated by neuronal activity at the cell membrane and propagated to the soma can unrestrictedly cross the nuclear envelope to invade the nucleus. The nuclear envelope, which allows ion transit via the nuclear pore complex, may represent a barrier for calcium and has been suggested to insulate the nucleus from activity-induced cytoplasmic calcium transients in some cell types.

**Results:**

Using laser-assisted uncaging of caged calcium compounds in defined sub-cellular domains, we show here that the nuclear compartment border does not represent a barrier for calcium signals in hippocampal neurons. Although passive diffusion of molecules between the cytosol and the nucleoplasm may be modulated through changes in conformational state of the nuclear pore complex, we found no evidence for a gating mechanism for calcium movement across the nuclear border.

**Conclusion:**

Thus, the nuclear envelope does not spatially restrict calcium transients to the somatic cytosol but allows calcium signals to freely enter the cell nucleus to trigger genomic events.

## Background

The compartmentalization of eukaryotic cells into membrane-delineated organelles spatially restricts molecules and allows specialized functions that require different biochemical microenvironments to be carried out simultaneously. To regulate and coordinate metabolic activities, mechanisms have evolved that allow information transfer across compartment borders. Many signaling pathways activated in the cytosol upon stimulation from the environment impinge on targets in the cell nucleus and regulate gene expression. In neurons, transcriptional responses induced by electrical activity are critical for long-lasting adaptive responses such as information storage, memory formation, or the activation of pro-survival programs [1-4]. Calcium is the principal second messenger that couples neuronal activity to gene regulation [5]. Several calcium-activated pathways can transmit signals to the nucleus. These include the ERK-MAP kinase and the p38 MAP kinase pathways, and a signaling pathway activated by the serine/threonine phosphatase, calcineurin (reviewed in [6]). However, the primary signal transducer is calcium itself that can propagate information from the site of signal generation at the plasma membrane into the nucleus [2,7]. Electrical activity-induced increases in the nuclear calcium concentration are required for CREB- and CBP-mediated gene expression [8-10]. Moreover, nuclear calcium signaling is critical for the long-lasting synaptic plasticity and learning [11], and induces the expression of a genomic pro-survival program [12,13]. Nuclear calcium transients in neurons are likely triggered by increases in the calcium concentration in the somatic cytosol. Although the nuclear envelope can restrict the exchange of molecules between the cytosol and the nucleoplasm, ions may enter and exit the nucleus via the nuclear pore complexes (NPCs). Precisely how nuclear calcium signals are generated and whether or not cytosolic calcium transients can freely cross the nuclear border is controversial [14,15]. In the mouse pituitary cell line, AtT20, and in a variety of primary neurons, electrical activity-induced somatic calcium signals appear to spread readily to the nucleus [8,10,16-18]. In contrast, in HeLa cells, neuroblastoma cells, and primary rat sensory neurons, the nucleus may be insulated from cytosolic calcium transients [19,20]. In addition, in Xenopus laevis oocytes, the filling state of intracellular calcium stores may regulate the conformational state of the NPC, which can affect diffusion of molecules between the cytosol and the nucleoplasm [21,22]. It is particular important to understand the dynamics of calcium signaling across the nucleo-cytoplasmic border in hippocampal neurons, where neuronal activity-induced nuclear calcium transients control a neuroprotective gene expression program [12,13] and are critical for learning and memory [11]. Here, we used laser-assisted photolysis of caged calcium compounds to investigate the properties of the nucleo-cytoplasmic exchange of calcium in hippocampal neurons. Our analysis revealed that calcium diffuses freely into and out of the nucleus with no apparent impediment at the nuclear envelope. We found no evidence for a gating mechanism for calcium movement through the NPC. Thus, in hippocampal neurons, calcium waves towards the cell soma do not face a detectable barrier at the nuclear compartment border.

## Results

### Release of calcium in spatially distinct regions using laser-assisted photolysis of caged calcium compounds

**Figure 1:**
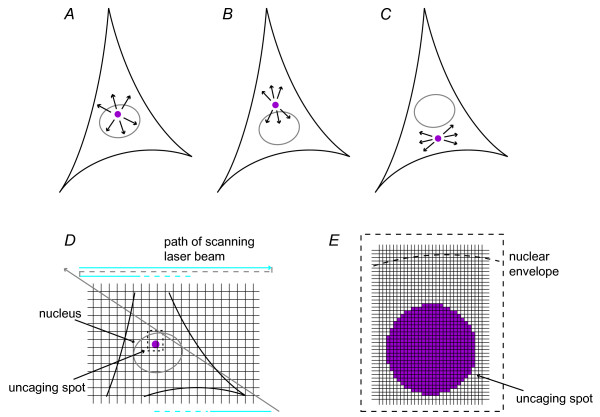
**Controlling calcium release in spatially distinct regions**. *A*, *B *and *C*, schematic illustration of the position of the area (pink circle) inside the nucleus or the cytoplasm that was illuminated with UV light for photolysis of NP-EGTA; arrows indicate the directions of calcium signal spreading analyzed. *D*, schematic illustration of image acquisition and calcium release that were performed simultaneously using a confocal laser-scanning microscope. The path of the laser beam (blue line) controlled by galvanometer driven scan mirrors starts in the upper left corner of the image and scans in the *x*-direction. At the end of the row the laser is switched off and the mirrors move back. The laser is switched on again and the scanning continues in the next row. This procedure is repeated up to the end of the image. The mirrors then move back to the starting position and the next image can be acquired. *E*, blow-up of the area containing the uncaging spot indicated in pink. For photolysis of NP-EGTA, the UV laser was switched on during the scanning process to illuminate the uncaging spot.

Hippocampal neurons were loaded with both the calcium indicator Fluo-4 and the calcium cage nitro-phenyl-EGTA (NP-EGTA) to release calcium in spatially distinct regions while monitoring calcium signals propagating throughout the cell (Figure 1A, B, and 1C). The local activation of NP-EGTA was achieved by exposure to ultraviolet (UV) laser light from a confocal laser scanning microscope. The scanning system allowed UV exposure with a rapid time course and high spatial precision, and thus produced calcium release in a defined area in a temporally controlled manner.

**Figure 2:**
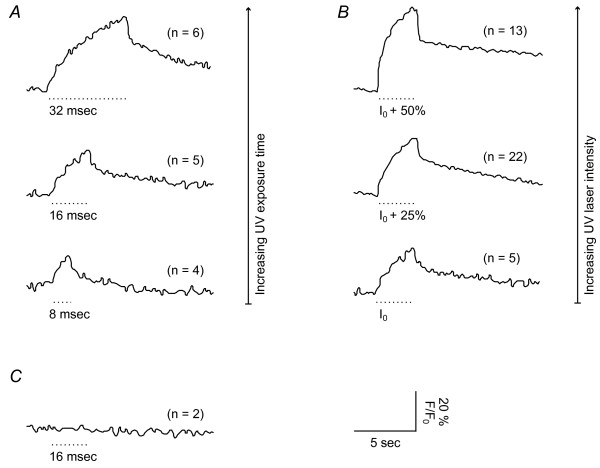
**Calcium release from NP-EGTA is dependent on exposure time and laser intensity**. *A *and *B*, neurons were loaded with NP-EGTA and Fluo-4. Uncaging of NP-EGTA was achieved with a continuous low level UV exposure over the whole scan frame. The characteristics of the calcium rise were controlled (*A*) by the exposure time (8, 16 and 32 msec, I_0 _= 0.5 mW) or (*B*) by the laser intensity (0.5, 0.63 and 0.75 mW, t_0 _= 16 msec). *C*, control cells were loaded with Fluo-4 only (I_0 _= 0.75 mW). The UV light was switched on for the indicated period (dotted line) although UV exposure occurred for only a fraction of each acquired frame (see Methods). The average of all cells (n) in the scan frame is shown. All traces represent normalized fluorescence of Fluo-4.

We first established the conditions to control calcium release. We exposed several cells in a field of view to UV light varying the exposure time (Figure 2A) as well as the UV laser power (Figure 2B). In both cases the calcium signal rose immediately after switching on the UV light. The amplitude of the calcium signal increased with the duration of the UV exposure; the rise time of the calcium transients decreased with the UV laser power. Furthermore, the amount of released calcium could be controlled by changing the area exposed to the UV light (data not shown). UV exposure to cells loaded only with Fluo-4 caused no change in the calcium concentration (Figure 2C). The UV exposure time was calculated taking into account that the activation of NP-EGTA occurred pixel by pixel over a period of several frames during the image acquisition (Figure 1D and 1E). Thus, the total exposure time *t *is calculated by *t *= *t*_*pixel *_× *n*_*pixel *_× *n*_*frame *_where *t*_*pixel *_is the time which is needed to scan one pixel, *n*_*pixel *_is the number of pixels of the uncaging area, and *n*_*frame *_the number of frames with UV exposure. The duration over which uncaging occurred *d *can be calculated by *d *= *n*_*frame*_/*freq*_*frame *_where *freq*_*frame *_is the imaging frequency.

**Figure 3:**
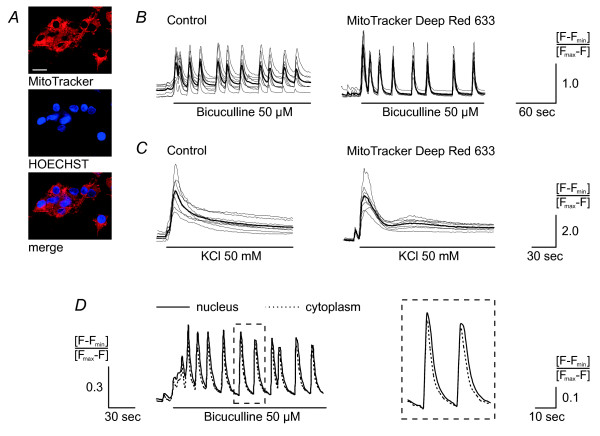
**Nuclear and cytoplasmic calcium signals are not affected by the mitochondrial dye**. *A*, neurons were incubated with the mitochondria-selective dye MitoTracker Deep Red 633 (red) and the DNA specific stain HOECHST 33258 (blue) for 20 min in SGG. Scale bar is 20 μm. *C *and *D*, no apparent differences in network properties were detected in hippocampal neurons loaded with Fluo-4 and MitoTracker Deep Red 633. Periodic calcium transients were induced by adding 50 μM of the GABA_A _receptor blocker bicuculline (*B*). Neurons were depolarized by exposing the cells to 50 mM KCl (*C*). Images were taken every 1.6 sec. Measurements of individual cells (thin grey lines) and their mean (bold black line) are shown. *D*, periodic calcium transients (induced by 50 μM bicuculline) in hippocampal neurons loaded with Fluo-4 and MitoTracker were measured in the nucleus (continuous line) and cytosol (dotted line). The average of n = 7 cells is shown. Image acquisition rate was 0.6 Hz.

We next selected a tool to precisely position the uncaging spot in the region of interest. Because we were interested in properties of calcium transients in different cellular compartments and calcium crossing the nuclear envelope (NE), we needed a dye that would allow us to distinguish nucleoplasm and cytoplasm. This dye should penetrate easily the plasma membrane without harming the cells and should not interfere with the uncaging procedure or calcium imaging. We chose the mitochondrion-selective stain MitoTracker Deep Red 633. Loading neurons with MitoTracker and the DNA specific stain HOECHST 33258 showed that MitoTracker was excluded from the nucleus (Figure 3A). For subsequent experiments, hippocampal neurons were loaded with MitoTracker, NP-EGTA, and Fluo-4. Calcium imaging experiments, in which hippocampal networks were either stimulated with the GABA_A _receptor blocker bicuculline (which triggers periodic and synchronous action potential bursting; [10,23]) or exposed to elevated extra-cellular K^+ ^concentrations (causing membrane depolarization), demonstrated that MitoTracker does not interfere with calcium signaling (Figure 3B and 3C). Closer examination of calcium signals induced by action potential firing revealed, at an image acquisition rate of 0.6 Hz, virtually synchronous time courses of the nuclear and cytoplasmic calcium transients (Figure 3D).

**Figure 4:**
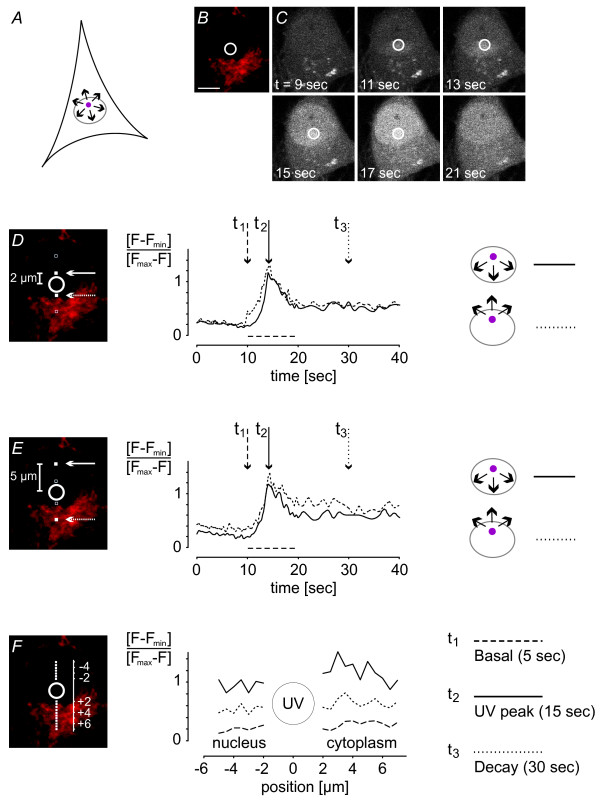
**Spreading of photolysis-induced nuclear calcium signals**. *A*, schematic illustration of the position of the 5 μm^2 ^area (pink circle) inside the nucleus that was illuminated with UV light for photolysis of NP-EGTA; arrows indicate possible directions of calcium signal spreading within the nucleus and across the NE to the cytoplasm. *B*, the positioning of the uncaging spot was guided by MitoTracker staining shown in red. *C*, time series of raw Fluo-4 fluorescence images of the calcium measurements shown in *D*, *E *and *F*; the indicated times correspond to the time scale of the graphs (*D *and *E*). The 5 μm^2 ^areas in the nucleus that were illuminated with UV light for photolysis of NP-EGTA are indicated with white circles. *D *and *E*, time courses of calcium transients were averaged in areas of 6 × 6 pixels (filled white squares) in the nucleus (continuous line) and the cytoplasm (dotted line) at 2 μm and 5 μm distances from the uncaging spot as indicated by arrows. UV exposure (*t*_*exp *_= 10.2 msec) occurred as indicated by the dashed line. Images were taken every 401 msec. *F*, the spatial profile of calcium signals at different time points (t_1 _= basal level, t_2 _= peak value, t_3 _= decay) is shown. Signals were measured as depicted in the mitochondria-stained image (filled white squares). The corresponding time points are indicated by arrows in the graphs (*D *and *E*). Scale bar is 5 μm.

**Figure 5:**
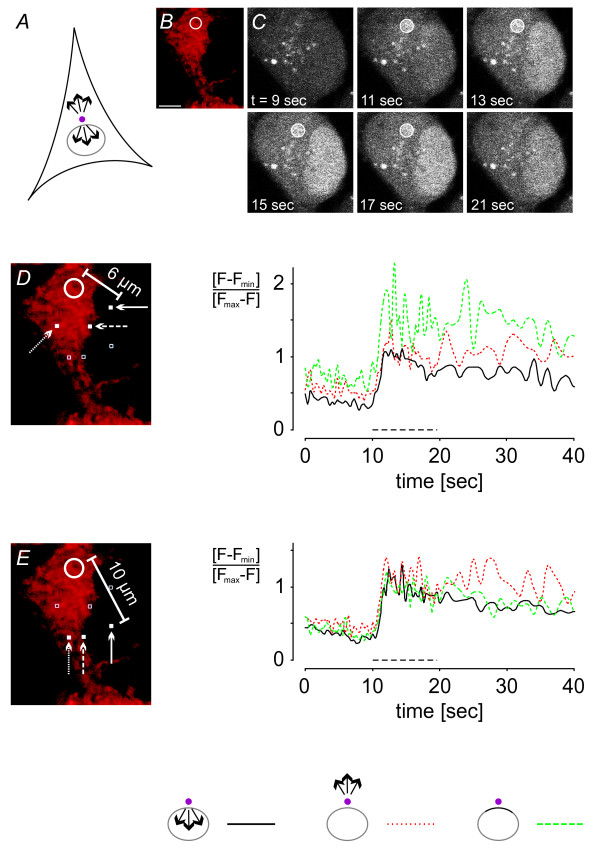
**Photolysis-induced cytosolic calcium elevations are followed by increases in nuclear calcium concentrations**. *A*, schematic illustration of the position of the 5 μm^2 ^area (pink circle) in the cytosol that was illuminated with UV light for photolysis of NP-EGTA; arrows indicate the directions of calcium signal spreading analyzed. *B*, the positioning of the uncaging spot was guided by MitoTracker staining shown in red. *C*, time series of raw Fluo-4 fluorescence images of the calcium measurements shown in *D *and *E*; the indicated times correspond to the time scale of the graphs. The 5 μm^2 ^areas in the cytoplasm that were illuminated with UV light for photolysis of NP-EGTA are indicated with white circles. *D *and *E*, time courses of calcium transients are shown at distances of 6 μm and 10 μm from the uncaging spot in the nucleus (continuous black line), in the cytoplasm (dotted red line), and at the perinuclear space (dashed green line); arrows point towards the positions of measurements (filled white squares). Images were taken every 401 msec. UV light was switched on as indicated by the dashed black line (*t*_*exp *_= 10.2 msec). Scale bar is 5 μm.

**Figure 8:**
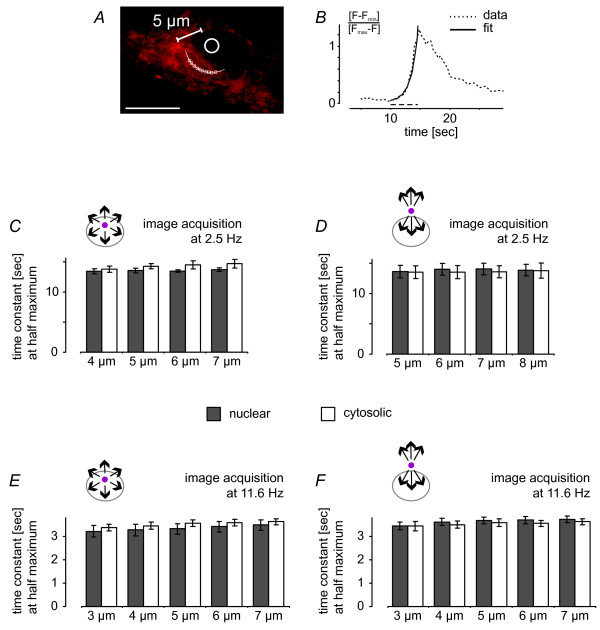
**Summary of the quantitative analysis of calcium signal spreading within a compartment and across the nuclear envelope**. Exponential curve fits were used to calculate the time constants at half maximum, *t*_*Fmax*/2_, for the calcium imaging traces measured at the various distances from the uncaging spot. *A *and *B*, an example of a mathematical fit and the corresponding experimental data is shown; in the example, the uncaging spot was localized to the nucleus and calcium was measured in the cytosol at a distance of 5 μm from the uncaging spot. Scale bar is 10 μm. *C *through *F*, summary of all *t*_*Fmax*/2 _values for calcium imaging traces measured within a compartment and across the nuclear envelope at the various distances from the uncaging spot; calcium imaging data were acquired at 2.5 Hz (*C *and *D*) and 11.6 Hz (*E *and *F*). Bars represent means ± s.e.m. (3 independent experiments; at each distance, 5 to 8 cells were analyzed in every experiment).

The cell loading procedure consisted of an incubation period with NP-EGTA for 90 min at 37°C and 5% CO_2_, followed by a 30-minute incubation period with the calcium indicator Fluo-4 acetoxymethyl (AM) ester at room temperature to avoid compartmentalization of the dye [24]. During subsequent deesterification of the AM ester the hippocampal neurons were treated with MitoTracker for a further 15 min. The loaded cells were mounted in a perfusion chamber and the uncaging spot was defined (Figure 4B and 5B). Calcium was released by UV light and Fluo-4 fluorescent changes were measured (Figure 4C and 5C). The total UV exposure time of an area of 5 μm^2 ^and 25 frames was ~10 msec. This was sufficient to evoke maximal calcium release from the caged compound. We considered four different conditions for our analysis of calcium propagation within cellular compartments and across the NE: uncaging in the nucleus and calcium diffusion both within the nucleus and across the NE (Figure 4); uncaging in the cytoplasm (near the nuclear border) and calcium diffusion both within the cytoplasm and across the NE (Figure 5). Calcium signals were measured at regions equidistant from the centre of the uncaging area (Figure 4D and 4E, Figure 5D and 5E). A summary of the quantitative analysis of all calcium imaging traces measured at the various distances from the uncaging spot within a compartment and across the nuclear envelope is shown in Figure 8.

### Generation and propagation of nuclear calcium signals

We first uncaged caged calcium in the nucleus. The signals were measured at a 2 μm and 5 μm distance from the uncaging spot inside and outside the nucleus. Irrespective of the presence of the nuclear border between the uncaging spot and the area of calcium measurements, the calcium signals rose with virtually identical slopes to the same maximum values (Figure 4D and 4E; see also summary of calcium imaging data in Figure 8C). To confirm that calcium diffused radially, we measured the signal at various points at 2 μm and 5 μm distances from the uncaging spot, determined the maximum values, and calculated by an exponential fit the time, *t*_*Fmax*/2_, required to reach half of the maximum value. The *t*_*Fmax*/2 _values obtained were: 13.4 ± 0.2 sec (n = 5) and 13.5 ± 0.2 sec (n = 5) in the nucleus at distances of 2 μm and 5 μm, respectively, from the uncaging spot, and 12.6 ± 0.1 sec (n = 5) and 13.7 ± 0.2 sec (n = 8) in the cytoplasm at distances of 2 μm and 5 μm, respectively, from the uncaging spot. The signal increases of the calibrated traces from basal to maximum were 0.92 ± 0.04 and 1.10 ± 0.13 at the 2 μm distance and 0.94 ± 0.08 and 0.89 ± 0.21 at the 5 μm distance for the nuclear and cytoplasmic signals, respectively. The differences in the values obtained for the 2 μm distance may be due to the close proximity of the region of calcium measurement and the site of calcium release. Because of the small size of the UV spot, a higher power of UV laser light (~2.4 mW) was required to obtain a sharp rise in calcium. It is conceivable that under those conditions, scattered light of the UV laser beam affected the calcium indicator in the immediate vicinity of the uncaging area. Visualizing the data in one-dimensional profiles at different time points confirmed that calcium spread equally throughout the cell. The calcium signals were similar on both sides of the NE at the resting ('basal') or the elevated state ('UV peak'). Even during the period of decay ('decay'), differences in the amplitudes of nucleoplasmic and cytoplasmic calcium signals could not be detected (Figure 4F). The virtually identical magnitudes and kinetics of signals at 2 μm and 5 μm as well as intermediate and larger diffusion distances (data not shown) indicate that calcium propagation out of the nucleus is not measurably reduced or slowed by the nuclear membrane.

### Generation and propagation to the nucleus of cytosolic calcium signals

It is possible that the process of calcium leaving the nucleus differs from that of calcium entering the nucleus. To test this, we shifted the uncaging spot to the cytosol and measured calcium propagation into the nucleus. To uncage amounts of calcium comparable to our nuclear uncaging experiments, we chose the same conditions (i.e. identical spot size, UV laser power and exposure time). The results were very similar to those obtained after nucleoplasmic calcium release. Signals were measured at a 6 μm and 10 μm distance from the centre of the uncaging spot in the cytoplasm and the nucleus. In addition, we monitored calcium signals at the border between nucleus and cytoplasm (Figure 5D and 5E; see also summary of calcium imaging data in Figure 8D). The nucleoplasmic signal increased as fast as the cytoplasmic signal and as fast as the signal in the perinuclear space at both the 6 μm and 10 μm distances (*t*_*Fmax*/2 _= 11.6 ± 0.3 sec (n = 17), 11.6 ± 0.3 sec (n = 13) and 11.5 ± 0.4 sec (n = 7) at the 6 μm distance for the cytoplasm, nuclear border and nucleus, respectively; *t*_*Fmax*/2 _= 11.7 ± 0.3 sec (n = 4), 11.8 ± 0.3 sec (n = 5) and 11.7 ± 0.2 sec (n = 9) at the 10 μm distance for the cytoplasm, nuclear border and nucleus, respectively). The peak calcium signal in the cytoplasm was slightly higher than that in the nucleus, whereas the maximum value in the perinuclear space was, at least at the 6 μm distance, higher than the cytoplasmic and nuclear values (0.95 ± 0.36, 1.74 ± 0.91 and 0.78 ± 0.17 at the 6 μm distance for the cytoplasm, nuclear border and nucleus, respectively; 0.95 ± 0.17, 0.75 ± 0.13 and 0.84 ± 0.12 at the 10 μm distance for the cytoplasm, nuclear border and nucleus, respectively).

**Figure 6:**
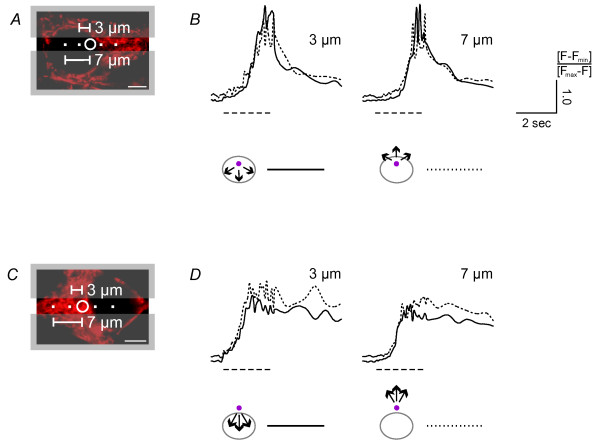
**Fast spreading of calcium signals within a compartment and across the nuclear envelope**. Cells were imaged within a narrow band to increase the image acquisition rate. *A *and *C*, the image frame was cut (indicated by grey boxes) to a field of view of 512 × 32 pixels. The 5 μm^2 ^areas in the nucleus (*A*) and in the cytosol (*C*) that were illuminated with UV light for photolysis of NP-EGTA are indicated with white circles; the positioning of the uncaging spot was guided by MitoTracker staining shown in red. The positions of calcium measurements at the 3 μm and 7 μm distances from the uncaging spot are indicated with white filled squares. *B *and *D*, calcium signals were determined using calibrated Fluo-4 fluorescence measurements in squares of 6 × 6 pixels at distances of 3 μm and 7 μm from the uncaging spot in the nucleus (continuous line) and the cytoplasm (dotted line). UV light (*t*_*exp *_= 10.0 msec) was switched on as indicated by the dashed line. Images were taken every 86 msec. Scale bar is 5 μm.

It remained possible that calcium measurements at a higher temporal resolution could reveal a partial barrier to calcium movement at the NE. We therefore repeated our experiments at higher frame rate (11.6 Hz) for both calcium signals propagating into and out of the nucleus. We achieved faster image acquisition by narrowing the field of view of the scanning system to 512 × 32 pixels (Figure 6A and 6C). Measurements of calcium signals at distances from the uncaging spot of 3 μm and 7 μm in the nucleus and the cytoplasm did not reveal any delay of calcium signals crossing the NE (Figure 6B and 6D; see also summary of all calcium imaging data in Figure 8E and 8F). For nuclear calcium release the *t*_*Fmax*/2 _values were 3.7 ± 0.2 sec (n = 3) in the nucleus and 3.7 ± 0.1 sec (n = 3) in the cytoplasm at a distance of 3 μm from the uncaging spot, and 4.0 ± 0.1 sec (n = 3) in the nucleus and 3.9 ± 0.1 sec (n = 3) in the cytoplasm at a distance of 7 μm from the uncaging spot. For cytoplasmic calcium release we obtained *t*_*Fmax*/2 _= 3.1 ± 0.1 sec (n = 3) in the nucleus and 3.3 ± 0.1 sec (n = 3) in the cytoplasm at a distance of 3 μm from the uncaging spot, and *t*_*Fmax*/2 _= 3.4 ± 0.1 sec (n = 3) in the nucleus and 3.4 ± 0.1 sec (n = 3) in the cytosol at a distance of 7 μm from the uncaging spot. A summary of all experiments performed at 11.6 Hz indicates that the kinetics of calcium signal propagation is independent of the direction of the calcium flow (i.e. into the nucleus vs. out of the nucleus) and not measurably affected by the presence of the NE (Figure 8E and 8F). Acquiring images at 11.6 Hz, we obtained similar results in experiments in which calcium signal propagation across the nuclear border was measured at 37°C (data not shown). Thus, calcium appears to diffuse freely between the cytosol and the nucleus and equilibrates quickly between the two compartments. Within the limits of the temporal resolution of our imaging experiments, the NE does not attenuate or slow down calcium movements and thus, does not represent a diffusion barrier for calcium in hippocampal neurons.

### Long-range calcium signaling within compartments and across the nuclear compartment border

**Figure 7:**
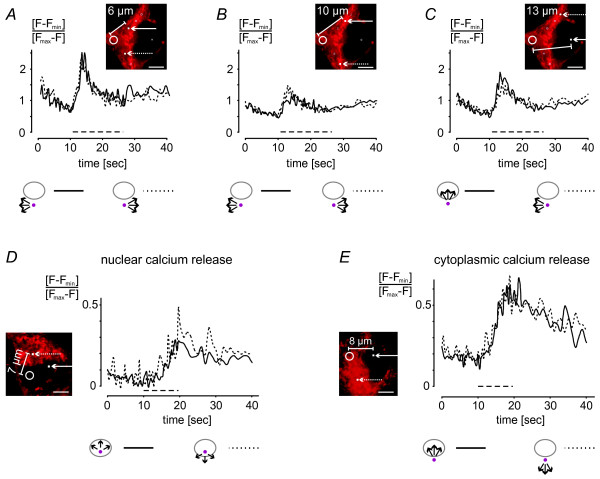
**Properties of calcium signal spreading are independent of the amounts of photolysis-induced calcium transients**. *A *through *E*, in the inserts, the 5 μm^2 ^areas that were illuminated with UV light for photolysis of NP-EGTA are indicated with white circles; the positioning of the uncaging spot was guided by MitoTracker staining shown in red. Arrows point towards the positions of calcium measurements at the indicated distances from the uncaging spot. Time courses of calcium transients were measured at the indicated distances in squares of 6 × 6 pixels (filled white squares). *A *and *B*, propagation of a photolysis-induced cytosolic calcium signal within the cytosol. *C*, propagation of a photolysis-induced cytosolic calcium signal within the cytosol and across the NE. *D *and *E*, propagation of photolysis-induced smaller size nuclear (*D*) or cytosolic (*E*) calcium signals within the compartment and across the NE. UV light was switched on as indicated by the dashed line (*A*, *B*, and *C*, *t*_*exp *_= 16.4 msec; *D *and *E*, *t*_*exp *_= 10.2 msec). Images were taken every 401 msec. Scale bar is 5 μm.

We further investigated possible differences between calcium signal spreading within one compartment and across the NE by placing the uncaging spot further away from the nucleus near a dendrite and measuring the cytoplasmic signal at several distances (Figure 7A and 7B). The time courses measured at 6 μm and 10 μm distances were virtually identical. We also acquired signals at a 13 μm distance from the cytosolic uncaging spot both in the nucleus and the cytoplasm (Figure 7C). The results obtained revealed that even at relatively distant regions the spreading of the calcium signal within a compartment and across the NE was similar. This property of calcium signal spreading appears to be independent of the calcium concentration and was also observed for smaller size photolysis-induced calcium transients (Figure 7D and 7E).

### The nuclear envelope is not a barrier for calcium signal propagation

A quantitative analysis of the time constants at half maximum, *t*_*Fmax*/2 _(calculated from exponential fits; see Figure 8A and 8B) from all imaging traces measured at the various distances from the uncaging spot confirmed that the presence of the NE in the path of calcium signal propagation does not lead to a measurable delay in signal spreading (Figure 8C, D, E and 8F). For calcium release in the nucleus acquiring images at 2.5 Hz, the time constants at half maximum, *t*_*Fmax*/2_, at the 5 μm distance from the uncaging spot measured in the nucleus and cytosol were 13.7 ± 0.4 sec (n = 3) and 14.3 ± 0.4 sec (n = 3), respectively; at the 7 μm distance from the uncaging spot they were 13.7 ± 0.3 sec (n = 3) and 14.7 ± 0.7 sec (n = 3) in the nucleus and cytosol, respectively. The time constants for calcium release in the cytosol measured at the 5 μm distance from the uncaging spot in the nucleus and cytosol were 13.6 ± 1.0 sec (n = 3) and 13.5 ± 1.0 sec (n = 3), respectively; at the 8 μm distance from the uncaging spot they were 13.9 ± 1.0 sec (n = 3) and 13.8 ± 1.3 sec (n = 3) in the nucleus and cytosol, respectively (Figure 8C and 8D). Acquiring images at 11.6 Hz, the time constants for calcium release in the nucleus measured at the 3 μm distance from the uncaging spot were 3.2 ± 0.4 sec (n = 3) and 3.4 ± 0.1 sec (n = 3) in the nucleus and cytosol, respectively; measured at the 7 μm distance from the uncaging spot, they were 3.5 ± 0.2 sec (n = 3) and 3.6 ± 0.1 sec (n = 3) in the nucleus and cytosol, respectively. For calcium release in the cytoplasm they were 3.4 ± 0.2 sec (n = 3) in the nucleus and 3.4 ± 0.2 sec (n = 3) in the cytoplasm at the 3 μm distance from the uncaging spot, and 3.7 ± 0.1 sec (n = 3) in the nucleus and 3.6 ± 0.1 sec (n = 3) in the cytoplasm at the 7 μm distance from the uncaging spot (Figure 8E and 8F). For both image acquisition rates (i.e. 2.5 Hz and 11.6 Hz), we obtained, for the various distances from the uncaging spot, virtually identical mean values of *t*_*Fmax*/2 _for calcium signal spreading within the compartment and calcium signal spreading across the NE (Figure 8C, D, E and 8F). The slightly wider distribution of the data obtained for calcium signals released in the cytoplasm may be due to a higher degree of variation of the uncaging-induced calcium events, which could be caused by calcium-induced-calcium-release (CICR) from intracellular calcium stores or by the uptake of calcium into intracellular stores.

### Lack of evidence for gating mechanism for calcium signal propagation across the nuclear border

**Figure 9:**
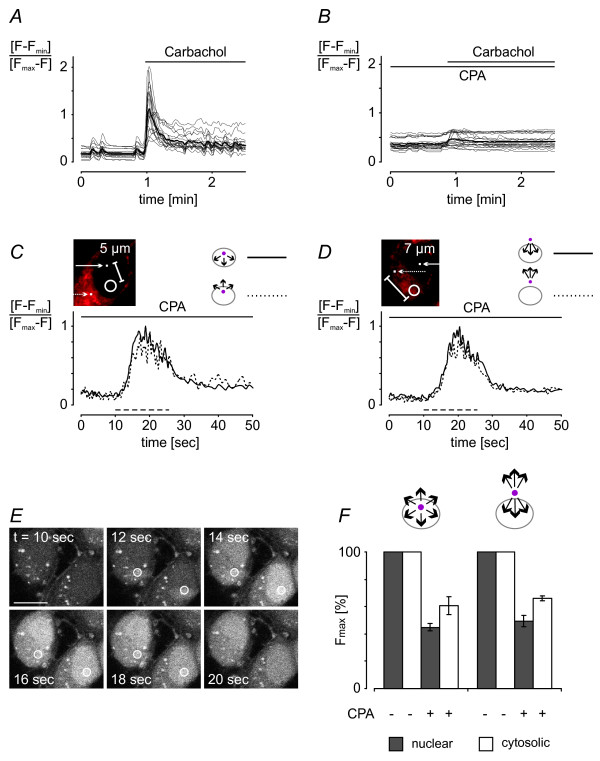
**Depletion of intracellular calcium stores did not alter calcium signal propagation across the nuclear envelope**. *A *and *B*, Fluo-4 calcium imaging experiments using hippocampal neurons stimulated with 100 μM of carbachol; carbachol-induced calcium rises (as shown in *A*) were abolished in hippocampal neurons pre-treated for 30 min with 30 μM cyclopiazonic acid (*B*). Images were taken every 1.6 sec. Measurements of individual cells (thin grey lines) and their mean (bold black line) are shown. *C *and *D*, propagation of photolysis-induced nuclear (*C*) or cytosolic (*D*) calcium signals within the compartment and across the NE in hippocampal neurons after CPA-mediated depletion of intracellular calcium stores. Calcium transients were measured at distances from the uncaging spot of 5 μm (*C*) and 7 μm (*D*) in the nucleus (*C *and *D*, continuous line) and the cytoplasm (*C *and *D*, dotted line). In the inserts, the 5 μm^2 ^areas in the nucleus (*C*) and in the cytosol (*D*) that were illuminated with UV light for photolysis of NP-EGTA are indicated with white circles; the positioning of the uncaging spot was guided by MitoTracker staining shown in red. Arrows point towards the positions of calcium measurements (filled white squares) at the indicated distances from the uncaging spot. UV exposure (*t*_*exp *_= 16.3 msec) occurred as indicated by the dashed lines. Images were taken every 401 msec. *E*, time series of raw Fluo-4 fluorescence images taken in the experiments shown in *C *(lower cell on the right) and *D *(upper cell on the left). The indicated times correspond to the time scale of the graphs (*C *and *D*). The 5 μm^2 ^areas in the nucleus and the cytoplasm, respectively, that were illuminated with UV light for photolysis of NP-EGTA are indicated with white circles. Scale bar is 10 μm. *F*, comparison of the maximum values (*F*_*max*_) of calcium rises in hippocampal neurons with and without CPA-mediated depletion of intracellular calcium stores. *F*_*max *_values were calculated using exponential curve fits for calcium imaging traces measured at distances of 4 μm, 5 μm, 6 μm, 7 μm, and 8 μm from the uncaging spot; *F*_*max *_values obtained for the various distances were pooled. Calcium imaging data were acquired at 2.5 Hz. Bars represent means ± s.d. (3 independent experiments; for each distance from the uncaging spot, 4 to 5 cells were analyzed in every experiment).

It has been suggested that diffusion of molecules across the NE is inhibited by the depletion of intracellular calcium stores [21,22,25]. We therefore investigated the possibility that calcium store depletion affects calcium movements into and out of the nucleus. ER calcium stores were emptied using the SR/ER calcium-ATPase inhibitor cyclopiazonic acid (CPA). Store depletion was confirmed by demonstrating that treatment of hippocampal neurons with the cholinergic agonist carbachol failed to generate calcium transients in CPA treated neurons (Figure 9A and 9B). Having depleted intracellular stores, we repeated our experiments of local uncaging to measure calcium diffusion from the nucleus towards the cytoplasm and vice versa. We found that upon UV light-induced calcium release in the nucleus or the cytoplasm, increases in calcium were detected on either site of the NE with virtually identical kinetics and amplitudes (Figure 9C, D, and 9E). For calcium release in the nucleus, the time constants *t*_*Fmax*/2 _measured at the 5 μm distance from the uncaging spot were 14.8 ± 0.2 sec (n = 9) and 15.1 ± 0.4 sec (n = 15) in the nucleus and in the cytoplasm, respectively (Figure 9C). For cytosolic calcium release, *t*_*Fmax*/2 _measured at the 7 μm distance from the uncaging spot were 16.5 ± 0.3 sec (n = 10) and 16.1 ± 0.4 sec (n = 9) in the nucleus and cytosol, respectively (Figure 9D). The rises of the calibrated traces from baseline to peak calcium signal in the nucleus and cytosol were 0.94 ± 0.09 and 0.77 ± 0.15, respectively, for calcium release in the nucleus and 0.85 ± 0.09 and 0.74 ± 0.16, respectively, for calcium release in the cytoplasm. Compared to control, the calcium signals obtained under conditions of calcium store depletion were smaller. For UV light-induced calcium release in the nucleus, the nuclear calcium and cytosolic calcium signals in CPA treated cells were 45 ± 3% (n = 4) and 61 ± 4% (n = 5), respectively, of those obtained in control conditions; for UV light-induced calcium release in the cytosol, the nuclear calcium and cytosolic calcium signals in CPA treated cells were 49 ± 7% (n = 4) and 66 ± 2% (n = 5), respectively, of those obtained in the control cells (Figure 9F). These results indicate that calcium release from intracellular stores contributes to the calcium transients observed following UV light-induced calcium release. However, calcium stores do not appear to be linked to a gating mechanism that could modulate calcium signal propagation across the nuclear border.

## Discussion

How signals cross intracellular compartment borders is a fundamental question in biology. In this study we analyzed the mechanism of calcium signal propagation across the nuclear envelope in hippocampal neurons.

### Nuclear calcium: key signal for adaptive responses in neurons

Neurons use changes in the intracellular calcium concentration to communicate signals generated by synaptic activity at the cell surface to the transcription-regulating machinery in the cell nucleus. Experiments in which either nuclear calcium was buffered [8] or the complex of calcium with calmodulin, the principal calcium sensor [26], was blocked specifically in the cell nucleus show that nuclear calcium signaling is a key mediator of neuronal gene expression [8-10], survival [12,13], and learning [11]. Given the central role of nuclear calcium in the control of adaptive responses, it is important to understand the precise mechanism of how calcium enters the cell nucleus and to uncover possible ways through which neurons can regulate this process to restrict access of calcium to the nucleus.

### Calcium signals freely cross the nuclear envelope in hippocampal neurons

There is evidence to suggest that the nucleus is insulated from large increases in the cytosolic calcium concentration [19,20]. The question arises as to what extend the nuclear membrane perforated by NPCs has an effect on particles of the size of around 0.2 nm, which corresponds to the atomic radius of calcium. NPCs are closely packed on the surface of the nuclear membrane [27]. Given the diameter of the central transporter region together with the spoke-ring complex of the NPC of approximately 50 to 100 nm [28,29] it is rather difficult to imagine how ion flux through the NPC could be restricted. A possible mechanism through which the cell could gate diffusion processes across the NPC has been suggested by Clapham and coworkers who identified by field emission scanning electron microscopy and atomic force microscopy a central plug in the NPC present in nuclear membrane preparations from Xenopus laevis oocyte [22]. Under condition where intracellular calcium stores are depleted, the plug occluded the NPC channel and blocked diffusion of intermediate size molecules (about 10 kDa), suggesting that calcium stores may regulate the conformational state of the nuclear pore complex, and thereby passive diffusion of molecules between the cytosol and the nucleoplasm. However, smaller molecules (such as Lucifer yellow) or ions were shown to diffuse freely across nuclear membrane preparation and intact nuclei even after calcium store depletion. Consistent with the observations in Xenopus laevis oocyte, we find that even after intracellular calcium store depletion, calcium can freely cross the nuclear border. These results do not rule out the existence of a central nuclear pore complex plug in hippocampal neurons; however, they suggest that a gating mechanism for calcium flux across the nuclear envelope does not exist.

### Possible other mechanisms for generating nuclear calcium signals

It remains an open question whether nuclear calcium transients can be generated without increases in the cytosolic calcium concentration. It is conceivable that calcium is directly released into the nucleoplasm from the inter-membrane space of the nuclear envelope, which is continuous with the endoplasmic reticulum (ER) [30,31]. Such a mechanism would depend on calcium release channels, such as the IP_3 _receptor or the ryanodine receptor being localized to the inner nuclear envelope. There are no electron microscopy studies that unambiguously demonstrate the presence of IP_3 _receptors and/or ryanodine receptors in the inner nuclear envelope. However, biochemical and electrophysiological studies using non-neuronal cells and Purkinje neurons suggested the both IP_3 _receptors and ryanodine receptors may be present in the inner nuclear envelope [32-36]. Moreover, CD38/ADP-ribosyl cyclase, the enzyme that catalyses the conversion of nicotinamide adenine dinucleotide to cyclic adenosine diphosphate ribose (cADP-ribose), an agonist of the ryanodine receptor, is localized to the inner nuclear envelope [37]. Ryanodine receptors, if present in the inner nuclear membrane and activated by cADP-ribose, could trigger calcium release into the nucleoplasm. However, to our knowledge signal-induced calcium transients that occur exclusively in the nucleus have not been observed in neurons. In addition, in HeLa cells, the analysis of spontaneous calcium events in over 700 cells failed to identify calcium signals that unambiguously originated in the cell nucleus [38], suggesting that HeLa cell nuclei are devoid of IP_3 _receptor- and/or ryanodine receptor-operated calcium stores. Our finding that photolysis-induced nuclear calcium transients are significantly smaller in hippocampal neurons after CPA-mediated calcium store depletion (see Figure 9F) are consistent with the existence of intra-nuclear sites of calcium release that may enhance nuclear calcium signaling. However, conditions under which hippocampal neurons generate nuclear calcium transients independently of cytosolic calcium increases remain to be identified.

## Conclusion

Calcium, the primary signal transducer in neuronal activity-dependent transcription, diffuses into and out of the nucleus with no apparent impediment at the nuclear envelope. The nuclear compartment border does not spatially restrict activity-induced calcium transients to the somatic cytosol but allows calcium signals to freely enter the cell nucleus to trigger genomic events.

## Methods

### Cell culture and stimulations

Hippocampal neurons from new-born Sprague-Dawley rats were prepared as described [39] except that growth media was supplemented with B-27 (Invitrogen, Karlsruhe, Germany). Cells were plated on poly-D-lysine and laminin-coated glass coverslips. Calcium imaging and uncaging were done after a culturing period of 10–14 days. Bursts of action potential firing were induced by treatment of cultured hippocampal neurons with 50 μM bicuculline (MP Biomedicals, Heidelberg, Germany). The membrane of hippocampal neurons was depolarized by increasing the extra-cellular K^+ ^concentration by 50 mM. Calcium release from intracellular stores was induced using bath application of 100 μM carbachol (Carbamylcholine chloride, Sigma-Aldrich Chemie GmbH, München, Germany). Depletion of intracellular calcium stores was achieved by treatment for 30 min with 30 μM cyclopiazonic acid (CPA, Tocris Bioscience, Bristol, UK), an inhibitor of the SR/ER Calcium-ATPase.

### Loading procedure, calcium imaging, and [Ca^2+^] measurements

Cells were loaded with 5–15 μM NP-EGTA, AM (Invitrogen, Karlsruhe, Germany) by incubation at 37°C, 5% CO_2 _for 90 min. Afterwards, they were loaded with 3.6 μM Fluo-4, AM (Invitrogen, Karlsruhe, Germany) by incubation at room temperature in darkness for 25 min. Before starting imaging/uncaging cells were loaded with 200 nM MitoTracker Deep Red 633 (Invitrogen, Karlsruhe, Germany). Fluorescence images were obtained using a Leica SP2 confocal microscope with an HCX PL APO CS 40.0 × 1.25 NA OIL UV objective (Leica, Wetzlar, Germany). Cells mounted in a perfusion chamber (LIS, Reinach, Switzerland) were imaged at room temperature in a buffered salt-glucose-glycine (SGG) solution containing (mM) NaCl 140.1, KCl 5.3, MgCl_2 _1.0, CaCl_2 _2.0, Hepes 10.0, glycine 1.0, glucose 30.0, and sodium pyruvate 0.5 on the stage of an inverted Leica DMIRBE microscope (Leica, Wetzlar, Germany). Experiments at higher frame rate (11.6 Hz) were also done at 37°C. Fluo-4 was excited by a 488 nm laser line and emission was collected at 500–550 nm. MitoTracker Deep Red 633 was excited by a 633 nm laser line and emitted light was collected at 640–720 nm. Release of calcium from NP-EGTA was achieved by exposure of predefined regions to UV laser light at 364 nm and 351 nm over a period of several frames during the image acquisition. The total exposure time *t *is calculated by *t *= *t*_*pixel *_× *n*_*pixel *_× *n*_*frame *_(*t*_*pixel *_= time to scan one pixel, *n*_*pixel *_= number of pixels of the uncaging area, *n*_*frame *_= number of frames with UV exposure). In the uncaging experiments, images were taken every 401 msec or 86 msec; in the experiments with bicuculline and carbachol, images were taken every 1.6 sec. To calibrate the fluorescence signal (*F*), Fluo-4 was saturated by adding 50 μM ionomycin (*F*_*max*_, Sigma-Aldrich Chemie GmbH, München, Germany) to the perfusion solution and then quenched with MnCl_2 _(*F*_*min*_). [Ca^2+^] was expressed as a function of the Fluo-4 fluorescence *K*_*d *_× [(*F-F*_*min*_)/(*F*_*max*_-*F*)] [40]. The validity of calcium measurements critically depends on the accuracy of the calibration, particularly when calcium concentrations are being analyzed and compared in different cellular compartments [41]. To avoid measurement artifacts, calibration was done pixel by pixel to relate the fluorescent values during calcium release with the correspondent values during the calibration. The time constants at half maximum, the *t*_*Fmax*/2 _values, were determined by approximating the data to an exponential function of the form *f(x) = exp(a+bx+cx*^2^). The coefficients *a*, *b *and *c *were calculated with the standard procedure of a mathematical fit based on the Gaussian method of least squares. All fits were performed considering the values during the rise of the calibrated traces from baseline to peak calcium signal.

## Authors' contributions

HB conceived of the study, and participated in its design and coordination and drafted the manuscript. AE participated in the study design and carried out all experiments described above. All authors read and approved the final manuscript.
